# SEA and GATOR 10 Years Later

**DOI:** 10.3390/cells10102689

**Published:** 2021-10-08

**Authors:** Yahir A. Loissell-Baltazar, Svetlana Dokudovskaya

**Affiliations:** CNRS UMR9018, Institut Gustave Roussy, Université Paris-Saclay, 94805 Villejuif, France; y.loissell@gmail.com

**Keywords:** SEA complex, GATOR complex, mTORC1 pathway, autophagy, amino acid signaling, cancer, epilepsy, neurological disorders

## Abstract

The SEA complex was described for the first time in yeast *Saccharomyces cerevisiae* ten years ago, and its human homologue GATOR complex two years later. During the past decade, many advances on the SEA/GATOR biology in different organisms have been made that allowed its role as an essential upstream regulator of the mTORC1 pathway to be defined. In this review, we describe these advances in relation to the identification of multiple functions of the SEA/GATOR complex in nutrient response and beyond and highlight the consequence of GATOR mutations in cancer and neurodegenerative diseases.

## 1. Introduction

The highly conserved mechanistic (or mammalian) target of rapamycin (mTOR) plays a key role in cellular homeostasis. mTOR kinase forms the following two different complexes: mTORC1 and mTORC2, which regulate cellular responses to many stresses [1,2]. In order to maintain optimal growth and metabolism, the mTORC1 pathway integrates signals from a wide variety of intracellular and extracellular cues, which include amino acids, growth factors, energy, oxygen, DNA damaging agents, etc. Depending on the nature of the signal, mTORC1 will drive the cell either to the anabolic pathway, promoting the proliferation and survival, or to the catabolic pathway by controlling autophagy or the ubiquitin-proteasome system. In order to coordinate this vast network, mTORC1 relies on many upstream modulators and downstream effectors. Ten years ago, one of the major upstream regulators of mTORC1 pathway, the SEA/GATOR complex, was identified [3]. Over these years, many advances have been made in our understanding of the SEA/GATOR complex functions and their consequences to the operation of the mTORC1 pathway; however, many questions are still unsolved [4]. Our comprehension of the SEA/GATOR complex regulation and function is particularly important because of the consequences of its dysfunction in diverse pathological settings, especially in cancer and neurodegenerative diseases. This review covers the most important findings about the SEA/GATOR complex that have been made during the last decade.

## 2. Discovery of the SEA Complex

The SEA complex was initially identified in yeast *Saccharomyces cerevisiae* through an atypical way [3,5,6,7]. Back in 2007, a multidisciplinary approach was undertaken to solve the structure of one of the largest macromolecular machines in the cell—the nuclear pore complex (NPC) [5,8]. Central to this approach were the collection of many kinds of biophysical and proteomic data, the translation of these data to spatial restrains and the calculation of a final architecture that satisfies all the restrains. This was how the immunopurification of one of the NPC components, nucleoporin Seh1, revealed that this protein did not only co-purify with the Nup84 subcomplex, the major constituent of the NPC scaffold, but also with the following four high-molecular-weight proteins with completely unknown functions at the time: Yjr138p (Iml1), Yol138p (Rtc1), Ydr128p (Mtc5) and Ybl104p [5]. Four years later, in 2011, a paper that described the full SEA complex for the first time was published [3]. The four proteins, which were first observed in Seh1 pullouts in 2007, were given a common name, Sea (for Seh1-associated) and named Sea1 through Sea4, respectively. The following three other protein components completed the full SEA eight-protein complex: Sec13, Npr2 and Npr3. The proteins of the SEA complex appeared to be dynamically associated with the vacuole membrane and have a role in autophagy. The function of Iml1-Npr2-Npr3 in autophagy was also described by the Tu group that same year [9]. Meanwhile, in 2009, Npr2 and Npr3 were shown to form an evolutionary conserved heterodimer, involved in the upstream regulation of TORC1 in response to amino acid starvation in *S. cerevisiae* [10]. This fundamental function of the SEA complex was further confirmed both in yeast and humans by de Virgilio and Sabatini laboratories in 2013 [11,12].

**Figure 1:**
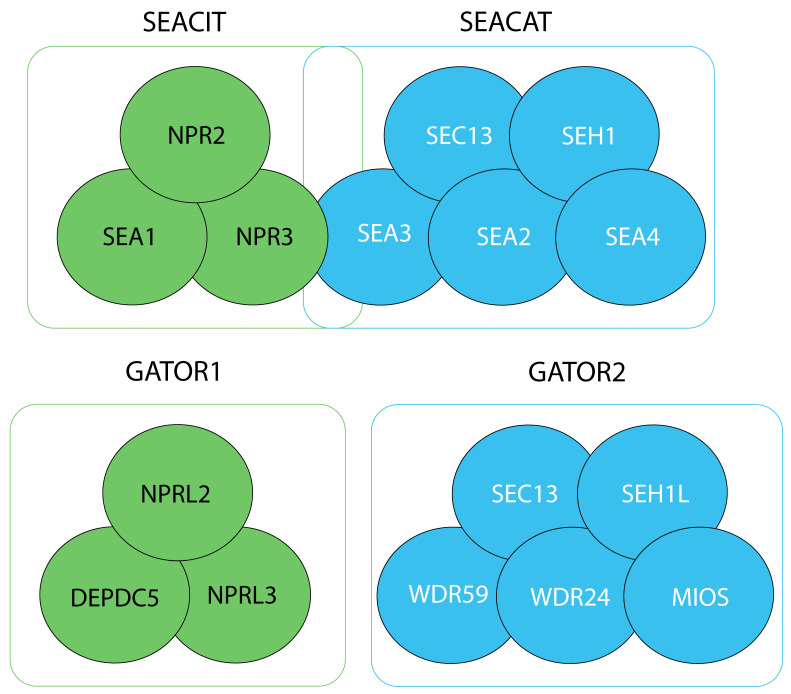
Composition of yeast SEA complex and mammalian GATOR complex. SEACIT subcomplex in yeast can tightly interact with SEACAT, most probably via Npr3/Sea3 connection. GATOR1 and GATOR2 do not form a stable full GATOR complex.

The SEA complex in *S. cerevisiae* consists of two subcomplexes, named SEACIT (SEA subcomplex inhibiting TORC1) and SEACAT (SEA subcomplex activating TORC1) (see below) [11,13,14] (Figure 1). In 2013, these complexes were characterized for the first time in humans by Sabatini’s laboratory and were re-named to GATOR1 (GTPase activating protein activity toward RAGA, see below) and GATOR2, respectively [12]. SEACIT is composed of Iml1/Sea1, Npr2 and Npr3 (DEPDC5, NPRL2 and NPRL3 in GATOR1), and SEACAT contains Sea2, Sea3, Sea4, Seh1 and Sec13 (WDR24, WDR59, MIOS, SEH1L, SEC13 in GATOR2) (Figure 1).

Phylogenetic analyses demonstrated that SEA/GATOR complex subunits are present across various eukaryotic kingdoms, suggesting an origin of these factors before the last common eukaryotic ancestor [3]. Homologs of all eight proteins could be clearly found in the genomes of fungi and metazoans, with some representation in protists, but cannot be identified in plants [3,15]. In 2021, the homologs of the SEA complex and its components were characterized in *Schizosaccharomyces pombe* [16], *Caernorhabditis elegans* [17], *Drosophila* [18], zebrafish [19], mice [20], rats [21] and humans [12]. The majority of the structural and functional studies were usually performed in *S. cerevisiae* and in humans. *Drosophila* was very instrumental for the study of the SEA/GATOR role in development; while the zebrafish, mouse and rat models were used to study different human pathologies.

## 3. SEA/GATOR Nomenclature

The nomenclature of the SEA complex proteins and subcomplexes in different organisms is somewhat confusing. For example, in *S. pombe*, the complex is called GATOR, but the names of the constituent proteins are the same as in *S. cerevisiae* [22]. One of the *Drosophila* GATOR1 components is called Iml1 (impaired minichromosome loss), as its yeast homologue, but all other proteins are named after their human homologues [18]. Moreover, the yeast protein community has a tendency to drop the name Sea1 and call the protein with its initial name, Iml1. The SEA proteins Npr2 (nitrogen permease regulator 2) and Npr3 (nitrogen permease regulator 3) gave names to their human orthologues NPRL2 (Npr2-like) and NPRL3 (Npr3-like) [10,23]. GATOR2 component MIOS obtained its name from its *Drosophila* orthologue Mio (missing oocyte) [24]. On the other hand, the two GATOR2 components, WDR24 and WDR59, still have their systematic names. In the future, it might be reasonable to revise their names so they reflect their function (currently, these functions are not yet defined). Alternatively, the proteins can be systematically named after their yeast homologues (as in the case of NPRL2, NPRL3), i.e., SEAL2 and SEAL3.

## 4. Structural Features of the SEA and GATOR Complexes

The overall architecture of SEA/GATOR proteins is evolutionary conserved [3]. DEPDC5 is only 10 amino acid residues longer than Iml1/Sea1, but both have an identical fold arrangement. The human orthologs of Sea2-Sea4, Npr2 and Npr3 are smaller than yeast proteins, mainly because of the deletion of protein regions, predicted to be disordered in yeast. It is quite reasonable to expect that the mammalian GATOR components repertoire would be larger compared to yeast due to the expression of alternative splicing products. For example, bioinformatical predictions revealed that WDR24 has at least two isoforms, one of which is missing about 130 amino acid residues in the N-terminal part [3]. One of the NPRL3 isoforms that lacks the N-terminal part and is highly expressed in red blood cells has just recently been characterized [25]. A splicing variant that led to exon 3 skipping in *NPRL2* was detected in an individual with familial focal epilepsy (see below) [26].

**Figure 2:**
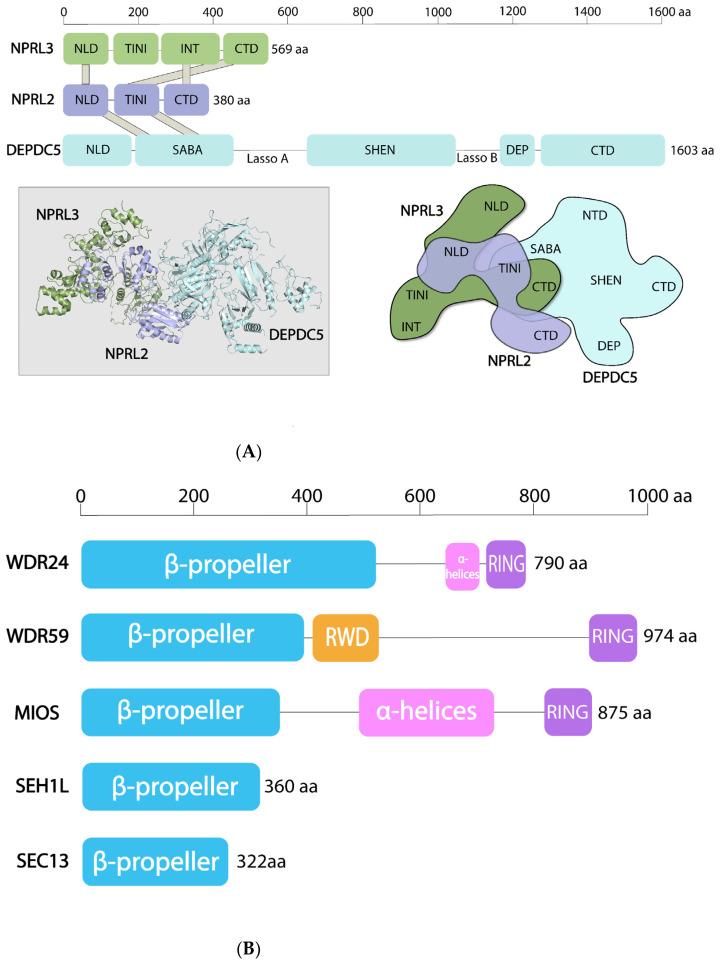
Domain organization of GATOR1 and GATOR2 proteins. (**A**) Domain structure and interaction of GATOR1 proteins (top); atomic model of GATOR1 complex (PDB:6CET), adapted from [29] and modified by PyMOL (bottom left) and cartoon representation of GATOR1 structure with domains indicated (bottom right). (**B**) Schematic representation of GATOR2 components, with domain boundaries according to secondary structure predictions from [3].

Two subcomplexes of the SEA/GATOR are very different structurally (Figure 2). SEACIT/GATOR1 members have domains, found in proteins that control the functions of small GTPases. SEACAT/GATOR2 components are enriched with domains found in coating assemblies (i.e., COPI and COPII coated vesicles, nuclear pore complex, etc.) (see below). Seh1, Sec13 and the N-termini of Sea4 and Sea2 in *S. cerevisiae* SEACAT appear to form a large cluster of β-propeller domains. Similar arrangements of β-propeller domains have been described at the vertex of the evolutionarily related complexes COPI and COPII [27].

In yeast, SEACAT and SEACIT interact to form the full SEA complex (Figure 1) [13]. A 3D map of the *S. cerevisiae* SEA complex, obtained by a combination of biochemical and computational approaches, suggests that SEACAT and SEACIT are connected by interactions between the N-termini of Sea3 from SEACAT and both Npr3 and Iml1/Sea1 from SEACIT [13]. Similar observations have recently been made in *S. pombe*, where Sea3 anchors other GATOR2 components to GATOR1, although, as expected, Sea3 was not required for the assembly of GATOR1 components [28]. In humans, GATOR1 and GATOR2 do not form a stable GATOR complex [12], yet, similar to yeast, NPRL3 is necessary and sufficient for the interaction with GATOR2 [29].

Despite the considerable progress in the structural determination of the constituents of the mTORC1 pathway that have been made in the last five years [30], only the structure of human GATOR1 has been solved (Figure 2A). All structural information that is currently available for GATOR2 or for the yeast SEA complex comes from bioinformatic predictions and interactivity assays [3,13]. The lack of high-resolution structures of the GATOR2 and of the entire complex both in yeast and humans are among the major reasons that prevent our full understanding of the SEA/GATOR functions at the present.

### 4.1. SEACAT/GATOR2

SEACAT and GATOR2 have components that moonlight between functionally unrelated complexes and are structurally connected with vesicle-coating scaffolds. The SEACAT/GATOR2 complex closely resembles the membrane coating assemblies, such as COPII vesicles, nuclear pore complexes and HOPS/CORVET complexes [8,31,32,33]. It also shares common subunits with both COPII (Sec13/SEC13) and nuclear pore complex (Sec13/SEC13 and Seh1/SEH1L). Sea4/MIOS contains N-terminal WD40 repeats arranged into a β-propeller structure followed by an α-solenoid stretch, which is a structure that is characteristic for proteins that form oligomeric coats (e.g., clathrin and Sec31) in vesicle-coating complexes [3,33] (Figure 2B). Furthermore, every protein in SEACAT contains a β-propeller (and Sea3 probably has two β-propellers), a domain common in coating assemblies [34]. Lastly, there are two dimers, Seh1-Sea4 and Sec13-Sea3 [3,13], that could be analogues to the Sec13-Sec31 dimer, which forms the structural unit of the COPII complex [35]. These dimeric interactions in the SEACAT are most probably conserved, because it was found that the Seh1 in *Drosophila* also directly interacts with Sea4/Mio [36].

Sea4 also contains a C-terminal RING domain, which, together with its β -propeller and α -solenoid motifs, makes it closely resemble several protein subunits of the homotypic fusion and protein sorting (HOPS) and class C core vacuole/endosome tethering (CORVET) complexes, which have been implicated in the tethering of membranes prior to their fusion. HOPS and CORVET are associated with the vacuoles/lysosomes and endosomes, respectively, and play a role in endosomal and vacuolar assembly and trafficking, as well as in nutrient transport and autophagy [32,37]. Sea2/WDR24 and Sea3/WDR59 also have a C-terminal RING domain. Clusters of RING domains are associated with E3 ubiquitin ligase activity, suggesting SEACAT might have such a role. In *S. cerevisiae*, the RING domains appear to be crucial for maintaining the interactions between Sea2, Sea3, Sea4 and the rest of the complex. For example, Sea4 that lacks the RING domain can only interact with Seh1, whereas Sea2 or Sea3 without the RING domain are no longer able to interact with any of the SEACAT complex components [13]. In addition, Sea3 contains an RWD domain that is enriched in β-sheets and common in proteins that also contain a RING motif and a β-propeller [38]. The RWD domain of Sea3 significantly resembles the RWD domain of the GCN2 protein, which is involved in general amino acid sensing and that of ubiquitin-conjugating E2 enzymes [39]. Given that SEACAT contains three proteins with RING domains, as well as numerous β-propeller domains that can mediate the recognition of phospho-substrate within E3 ligase complexes [40], it will be very interesting to investigate whether SEACAT/GATOR2 can act as a E3 ubiquitin ligase, and if this is the case, what are its possible targets.

The presence of the same folds and fold arrangements in both the SEA complex and in coating and tethering assemblies, and the fact that they contain the same “moonlighting” components, are suggestive that these complexes share a common evolutionary origin (see below). The majority of intracellular membranes are likely a result of the evolutionary expansion of an ancestral membrane-curving module—termed the “protocoatomer” complex [31,34]. The SEA complex is a member of the coatomer group, and its existence, thus, provides further evidence that an expansion of the protocoatomer family underpins much of the functional diversity of the endomembrane system.

### 4.2. SEACIT/GATOR1

The structural profile of the SEACIT/GATOR1 subunits is completely different (Figure 2A). Npr2/NPRL2 is a paralog of Npr3/NPRL3 [10,41] and both proteins possess N-terminal longin domains [42,43]. Iml1/Sea1 and its human homologue DEPDC5 contain a unique composition of domains that are not found in any other proteins. SEACIT components also have PEST motifs that often exist in rapidly degraded proteins [3]. However, PEST motifs are not well preserved in mammalian orthologues and, thus, could be a specific feature of the yeast SEA complex.

The structure of GATOR1, resolved recently by cryo-EM, revealed the architecture of each GATOR1 component [29] (Figure 2A). DEPDC5 has the following five defined domains: N-terminal domain (NTD), followed by SABA (structural axis for binding arrangement), SHEN (steric hinderance for enhancement of nucleotidase activity), DEP (Dishevelled, Egl-10 and Plekstrin) and C-terminal (CTD) domains. Interestingly, NTD, SABA and DEP domains can be found in membrane-associated proteins. For example, a domain similar to NTD exists in the SNARE chaperone Sec18/NSF, the SABA domain—in Sec23 of COPII vesicles (again returning to the theme of coating complexes). The DEP domain, which has diverse functions in signal transduction, is involved in the interactions between the regulator of G protein signaling (RGS) proteins and their membrane-bound receptors, the GPCRs [44]. The DEP domain is also found in a DEPTOR subunit of mTORC1 [45].

NPRL2 and NPRL3 have a similar structure with N-terminal longin domains that heterodimerize (Figure 2A). C-terminal domains of NPRL2 and NPRL3 also form a large contact surface. The SABA domain in DEPDC5 interacts with the NPRL2 TINI domain (tiny intermediary of NPRL2 that interacts (with DEPDC5)). By the way, the domain nomenclature within the GATOR1 complex created a doubtful precedent, where protein domains are named after the first (SHEN) and the last (SABA-TINI) authors of the article that reported the structure [29].

### 4.3. Posttranslational Modifications of SEA/GATOR

The majority of the information about post-translational modifications of the SEA/GATOR components came from whole proteome studies, essentially in yeast [46,47,48,49,50,51]. All the SEA and GATOR members are heavily phosphorylated and ubiquitinated (except of Sec13), with many modifications occurring at the disordered regions of proteins. However, there are still very few studies that explore the functional role of these modifications. Several papers, which describe the effect of ubiquitination, are manly focused on the role of this modification on protein stability. Thus, Npr2 in yeast interacts with Grr1, the F-box component of the SCF^Grr1^ E3 ubiquitin ligase [52]. Moderately unstable Npr2 is stabilized in *grr1Δ* mutants. In response to amino acids, CUL3-KLH22 E3 ubiquitin ligase induces K48 polyubiquitination on multiple DEPDC5 sites leading to its degradation [53]. Accordingly, DEPDC5 levels are increased during amino acid starvation. In the rich media, NPRL3 is more resistant to proteasome degradation than NPRL2 [54]. The data about the stability of SEA/GATOR proteins during amino acid starvation are contradictory and vary considerably in different species. For example, the level of practically all the SEA members in yeast decreases both during amino acid starvation and rapamycin treatment [13]. In *Drosophila* S2 cell lines, amino acid deprivation increases Nprl3 stability [55], although the reports in human cell lines indicate that the amount of NPRL2 and NPRL3 is not changed at least after 30 min of amino acid starvation [53]. It is reasonable to expect in the following years that we will gain more information about the role of posttranslational modifications not only on the stability of SEA/GATOR members, but also on their function.

## 5. Function of the SEA and GATOR in Nutrient Sensing and Responding

### 5.1. Overview of Amino Acid Axis of Signaling to mTORC1

**Figure 3:**
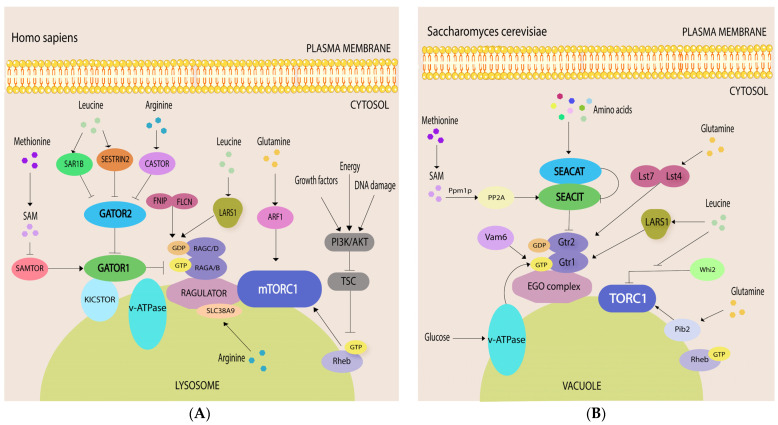
Amino acid signaling. (**A**) mTORC1 signaling in *Homo sapiens*. (**B**) TORC1 signaling in *Saccharomyces cerevisiae*. Yeast and mammalian orthologues are designated with the same color. Arrows and bars represent activation and inhibition, respectively. See text for more details.

One of the principal roles of SEA and GATOR as upstream regulators of mTORC1 is responding to amino acid availability [11,12] (Figure 3), although the role of both GATOR subcomplexes in glucose sensing has also been reported recently [56]. Effective functioning of the mTORC1 pathway with respect to cellular amino acid levels requires coordinated action of RAG guanosine triphosphatases (RAG-GTPases or RAGs) and their effectors, such as GTPase-activating proteins (GAPs), which stimulate GTP hydrolysis and guanine-nucleotide-exchange factors (GEFs). The major site of mTORC1 activation is the vacuole/lysosomal surface, where mTORC1 is recruited and induced in an RAG-GTPase dependent manner when amino acids are abundant [57,58]. There are the following four RAG GTPases: RAGA and functionally redundant RAGB; RAGC and functionally redundant RAGD (Figure 3A). They exist as obligate heterodimers, e.g., RAGA (or RAGB) with RAGC (or RAGD). RAGs interact with a pentameric RAGULATOR complex, anchored to the lysosome [57,58,59,60,61]. RAGULATOR also interacts with v-ATPase, a protein pump at the lysosomal membrane. The guanine nucleotide loading is important for RAGs function. In the presence of amino acids, RAGs are active when RAGA/B is loaded with GTP, and RAGC/D is bound to GDP. Reversely, when amino acids are low, RAGs are inactive, and RAGA/B is loaded with GDP and RAGC/D is bound to GTP. Various GAPs and GEFs promote the conversion of RAGs from active to inactive form. This is where the SEACIT and GATOR1 complexes exert their major functions (see below). A RAG-independent induction of mTORC1 by amino acids both at the vacuole/lysosome and Golgi has also been described in yeast and humans [62,63,64,65], but will not be thoroughly discussed in this review since neither SEA nor GATOR seem to be involved in this mode of mTORC1 activation. Moreover, a recent study revealed that RAG-independent activation of mTORC1 by amino acids derived from protein degradation in lysosomes required HOPS complex and was negatively regulated by activation of the GATOR-RAGs pathway [37]. Thus, evolutionary related HOPS and GATOR2 [3] have similar but divergent roles in activating mTORC1 in response to different amino acid inputs.

When amino acids are scarce, some amino acid sensors (see below) interact with and inhibit the GATOR2 complex, thus preventing inhibition of the GATOR1 by GATOR2 (Figure 3A). A mammalian-specific KICSTOR complex tethers GATOR1 to the lysosomal surface [66,67] where GATOR1 acts as a GAP for RAGA [12], thereby transforming RAGA to its inactive, GDP bound form, which further leads to mTORC1 suppression (Figure 3A).

In the presence of amino acids, RAGULATOR and v-ATPase undergo a conformational change that results in RAGULATOR exerting GEF activity towards RAGA or RAGB [60]. RAGULATOR can also trigger GTP release from RAGC [68]. In parallel, upon arginine binding arginine sensor SLC38A9, which resides at the lysosome, stimulates GDP release from RAGA [68]. A complex between folliculin (FLCN) and folliculin-interacting protein (FNIP) 1 and/or 2 is a GAP for RAGC/D [69]. In addition, leucyl-tRNA synthetase (LeuRS or LARS1 or LRS) also has GAP activity towards RAGD [70]. Active RAGULATOR-RAG stimulates recruitment of mTORC1 to the lysosomal membrane where it is fully activated by small GTPase, RHEB, loaded with GTP [71]. RHEB is under the control of another signaling node—the TSC complex, composed of TSC1, TSC2 and TBC1D7, where TSC2 acts as a GAP to inhibit RHEB. TSC is a nexus of multiple physiological stimuli (e.g., energy status, growth factors, DNA damage) that signal to mTORC1 through PI3K-AKT network [72]. RAG GTPases regulate the recruitment of TSC to the lysosome and its ability to interact with and inhibit RHEB in response to amino acid starvation, growth factors removal and to other stresses that inhibit mTORC1 [73,74,75]. Both RAGs and RHEB are necessary for mTORC1 activation at the lysosome, as the lone presence of either one is not sufficient. Accordingly, only when both the RAG GTPases and RHEB are inactive mTORC1 fully released from the lysosome [73].

The RAGs and RAGULATOR are conserved both in fission and in budding yeast (Figure 3B) [76,77]. Thus, the orthologue of RAGA/B, a protein called Gtr1 in yeast, forms a heterodimer with Gtr2, which is an orthologue of RAGC/D. Similar to mammals, in order to activate TORC1, GTP-bound Gtr1 and GDP-loaded Gtr2 interact with trimeric *S. cerevisiae* Ego1-Ego3 complex (Lam1-Lam4 in fission yeast) analog of RAGULATOR. Iml1/Sea1 from the SEACIT serves as a GAP for Gtr1 in the absence of amino acids [11]. Interestingly, LARS1 in yeast is the GEF for Gtr1 [78], while in mammalian cells LARS1 was shown to be a GAP for RAGD [70], although a GAP activity was not confirmed in a later study from different laboratory [69]. Lst4-Lst7 complex, an orthologue of mammalian FLCN/FNIP, is GAP for Gtr2 [79]. The GEF for Gtr2 in yeast and for RAGC/D in mammals is still not known.

There are some notable differences between yeast and humans during amino acid signaling to mTORC1 (Figure 3). First, many amino acid sensors (e.g., SAMTOR, SESTRINs) are absent in yeast (see below) [1]. Second, v-ATPase in yeast, which interacts with Gtr1, seems to activate TORC1 in response to glucose [80]. Third, RHEB orthologue in yeast *S. cerevisiae* seems not to be involved in TORC1 signaling, although it is required for arginine and lysine uptake [81]. Fourth, *S. cerevisiae* does not have TSC homologues, thus the entire branch of TSC/RHEB signaling is not conserved in this particular yeast. In contrast, *S. pombe* has both RHEB and TSC, which are involved in mTORC1 activation. How *S. cerevisiae* achieves full TORC1 activation at the vacuole without TSC/RHEB branch is currently not well understood.

### 5.2. GATOR2 Interactions with Leucine Sensors SESTRINs and SAR1B and Arginine Sensor CASTOR1

Cytosolic leucine can be sensed by the proteins from the SESTRIN family (SESTRINs 1–3) [82,83,84], by small GTPase SAR1B [85] and by leucyl-tRNA synthetase [70,86,87]. Arginine is sensed by CASTOR1 protein homodimer in the cytoplasm [88,89] and by SLC38A9 together with TM4S5F protein at the lysosomal membrane [90,91,92].

GATORs can interact directly with several amino acid sensors (Figure 3A). During leucine or arginine starvation, SESTRIN2 [82], SAR1B [85] or CASTOR1, respectively [88,89] interact with and inhibit the GATOR2 complex. WDR24 and SEH1L are essential for interaction with SESTRIN2, but it is not known which component of GATOR2 interacts with SESTRIN2 directly [93,94]. SAR1B directly binds MIOS, but not other GATOR2 subunits [85]. WDR24, SEH1L and MIOS were sufficient for interaction with CASTOR1 [89]; the CASTOR1 N-terminal domain is involved into direct interaction with MIOS [95]. Binding sites for SESTRIN2 and CASTOR1 are located at different parts of GATOR2 [89]. These interactions prevent inhibition of the GATOR1 by GATOR2 [96] and as a consequence, lead to mTORC1 inhibition. Neither SESTRIN2 nor CASTOR1 interact with GATOR1 [89,93,97].

In the presence of amino acids, interaction of leucine to the defined binding pocket in monomeric SESTRIN2 [83] or arginine with its binding pocket at the homodimeric CASTOR1 [88,95,98] results in dissociations of these sensors from GATOR2 and relieves mTORC1 inhibition. It is important to note, however, that SESTRIN2-GATOR2 interactions were initially observed in the cell-lines cultured in leucine-rich conditions [93,97], even if amino acid starvation enhanced this interaction. In vitro addition of leucine reduces the SESTRIN1-GATOR2 or SESTRIN2-GATOR2 interactions, but it does not affect SESTRIN3-GATOR2 interaction [82,84]. Interestingly, SESTRIN2 and SAR1B detect different parts of leucine; SAR1B recognizes the amino group and side chain of leucine [85], while SESTRIN2 interacts with leucine’s amino and carboxyl groups [83].

Interactions of SESTRINs to GATOR2 depends on a cell type and physiological conditions. Thus, in the skeletal muscle of rats, SESTRIN1 is the most abundant isoform, and SESTRIN2 expression is much lower relative to either SESTRIN1 or SESTRIN3. Accordingly, oral administration of leucine to fasted rats induced SESTRIN1-GATOR2 disassembly, but did not affect the interaction of other SESTRIN isoforms with GATOR2 [84]. This suggests that in the rat skeletal muscle, it is probably SESTRIN1 that has a primary role as a leucine sensor and leucine-induced activation of mTORC1 in skeletal muscle happens via SESTRIN1 release from GATOR2. SESTRINs–GATOR2 interactions can also be age dependent. Thus, in the skeletal muscle of young pigs, SESTRIN2 is more abundant than SESTRIN1 but the GATOR2 amounts are the same. Accordingly, during amino acid starvation the abundance of the SESTRIN2–GATOR2 complex reduced more in younger pigs [99].

Recently, GATOR2 was reported to be required for SESTRIN2-induced AKT activation and AKT translocation to plasma membrane [94]. In addition, GATOR2 physically bridges SESTRIN2 with mTORC2 where WDR59’s interaction with mTORC2’s component RICTOR is essential for the communication between GATOR2 and mTORC2, and WDR24 is crucial for GATOR2-SESTRIN2 interaction. In HeLa cells, GATOR2 promotes AKT activation and facilitates AKT-dependent inhibitory phosphorylation of TSC2 [75]. Thus, although an exact molecular function of GATOR2 has not yet been defined, it is clear that GATOR2 might have a large repertoire of various activities. Solving the structure of GATOR2 alone and in complex with its interactors will provide essential information about how these multiple functions can be exerted.

### 5.3. GATOR1 Interaction with SAM Sensor, SAMTOR

The SAM sensor, SAMTOR, binds to GATOR1 during SAM or methionine deprivation, and negatively regulates mTORC1 activity [100]. The component of GATOR1 that interacts with SAMTOR is currently unknown. In the presence of SAM, this metabolite occupies its binding pocket in SAMTOR, which disrupts the interaction of an amino acid sensor with GATOR1, promoting mTORC1 activity. SAMTOR and GATOR1 interactions are dependent on KICSTOR (see below). When SAMTOR is bound to SAM, it dissociates from GATOR1–KICSTOR, thus inhibiting GATOR1 and promoting mTORC1 activation [101]. On the other hand, methionine starvation promotes interaction between SAMTOR and the GATOR1–KICKSTOR complex, but weakened the interaction between GATOR1 and GATOR2, thus leading to mTORC1 suppression [100]. SAM levels can be affected by the availability of vitamin B12. Mice NPRL2 KO embryos have significantly reduced methionine levels and demonstrate phenotypes reminiscent of B12 deficiency [20]. It is currently unknown whether methionine can be sensed directly. Interestingly, leucine can also signal to mTORC1 through its metabolite, acetyl-coenzyme A, but in a RAG-independent and cell-specific manner [102].

In a recent study, Jewell laboratory investigated the potency of each amino acid to stimulate mTORC1 in MEF or HEK293 cells [65]. Ten amino acids were able to re-stimulate mTORC1 and promote its lysosomal localization. Glutamine and asparagine signal to mTORC1 through a RAG-independent mechanism via ADP-ribosylation factor ARF1. Eight amino acids (alanine, arginine, histidine, leucine, methionine, serine, threonine and valine) filter through RAGs. While three cytoplasmic sensors for leucine, arginine and methionine (SAM) have been identified, it is not known whether the other five amino acids also have their specific sensors and whether they will interact with GATORs.

### 5.4. SEACIT and Amino Acid Sensing in Yeast

Amino acid sensing in yeast differs significantly from the mammalian system (Figure 3B). SESTRINs, CASTOR1 and SAMTOR are not conserved in *S. cerevisiae* and *S. pombe*, which presumes that the interaction of these amino acid sensors with GATOR complexes arose later in the evolution. Nevertheless, Npr2 does participate in methionine sensing in *S. cerevisiae*, but in a very different way than in mammals. Under normal growth conditions, Ppm1p methyltransferase methylates two subunits of yeast protein phosphatase 2A (PP2A), which promotes Nrp2 dephosphorylation, TORC1 activation and suppression of autophagy [103]. Low methionine level leads to a decreased SAM, which blocks PP2A methylation and its phosphatase activity. As a result, Npr2 accumulates in phosphorylated form, which most probably changes the integrity of the SEACIT complex due to increased interaction between phosphorylated Npr2 and Iml1/Sea1 [9]. Therefore, SEACIT is no longer able to repress TORC1 effectively, resulting in autophagy activation. Interestingly, Npr2-deficient yeast grown in a minimal medium, containing ammonium as a sole nitrogen source and lactate as a nonfermentable carbon source, metabolize glutamine into nitrogen-containing metabolites and maintain high SAM concentrations [104].

As in mammals, yeast also have amino acid sensing pathways parallel to SEA-GTR signaling [105]. For example, Pib2, which resides at the vacuole membrane, interacts with TORC1 complex in a glutamine-sensitive manner, suggesting that Pib2 acts as a part of a putative glutamine sensor [64]. Although both Pib2 and EGO are required for TORC1 tethering to the vacuolar membrane and its activation, they form different complexes with TORC1, ruling out a possibility that the SEA complex can participate in Pib2-dependent amino acid sensing. Even if Pib2 does not have apparent ortholog in mammals, PLEKHF1 protein shares high sequence similarity with Pib2 domains, important for TORC1 activation. However, PLEKHF1 is not involved in the glutamine-dependent regulation of mTORC1 [65]. In addition, Whi2, localized at the cell periphery, specifically senses low amino acid levels in general and leucine levels in particular, and suppresses TORC1 activity independently of the SEA complex [106,107]. The Whi2 homologue in mammals, KCTD11, acts as a negative regulator of mTORC1 during amino acid deprivation [106].

All these recent findings demonstrate that amino acid sensing mechanisms are way more diverse, because not only amino acids themselves, but also their metabolites can be sensed in a RAG-dependent, RAG-independent and cell-specific manner.

Many questions about amino acid sensing ultimately related to SEA and GATOR functions remain unanswered. Does every amino acid have its own sensor? Will all the sensors that work through RAGs interact with GATORs? What are the determinants of the interaction of amino acid sensors with one or another GATOR complex? In other words, why do SESTRIN2 and CASTOR1 interact with GATOR2, and SAMTOR with GATOR1? What are the factors that determine sensing of the same amino acid by different sensors? For example, why does leucine need three sensors (SESTRIN2, SAR1B and LARS1) that function in the same cell types, in the same subcellular location (cytosol), through the same pathway (RAG-dependent)? Leucine can also signal through its catabolite acetyl-CoA and activate mTORC1 via EP-300-mediated acetylation of RAPTOR [102]. Can other amino acids signal both themselves and their metabolites through different sensors? For example, the methionine metabolite SAM is sensed by SAMTOR, does a methionine sensor exist? Amino acid sensing also happens at Golgi, where GATORs, SESTRINS, CASTOR1 and SAMTOR have not been found thus far. How is amino acid sensing is achieved at Golgi? What is the repertoire of cell-type specific sensors? The primary role of aminoacyl tRNA synthetases is binding to cognate amino acids and their attachment onto appropriate tRNAs. Some of them, such as cytosolic LARS1 [70,78] and mitochondrial TARS2 (but not cytosolic TARS) [108], are also implicated in the upstream regulation of mTORC1 pathway. Are other aminoacyl tRNA synthetases also involved in mTORC1 regulation? What are the details of a crosstalk between general amino acid sensing through GCN2 and sensing through the mTORC1 pathway? Finally, what are the main determinants of amino acid sensing in yeast given that many mammalian amino acid sensors discovered thus far do not have yeast homologous, yet the GATOR-RAG-RAGULATOR (SEA-GTR-EGO) system is conserved?

### 5.5. SEACIT/GATOR1 as GAP for EGO/RAG

Two papers published simultaneously in 2013 reported the results that have dramatically increased the significance of the SEA/GATOR complex in the regulation of mTORC1 pathway. The laboratory of Claudio de Virgilio found that in *S. cerevisiae*, the SEA subcomplex, which was subsequently named SEACIT (SEAC subcomplex inhibiting TORC1 signaling) [14], acts as a GAP for Gtr1 and, thus, inhibits TORC1 [11]. In a parallel study, David Sabatini’s laboratory characterized for the first time the human homologue of the SEA complex, and also found the GAP activity of the SEACIT analogue, which received the GATOR1 name (GTPase activating protein activity towards RAGA) [12]. In both yeast and humans, SEACAT/GATOR2 acts upstream of SEACIT/GATOR1, suppressing its GAP activity, thus being “an inhibitor of an inhibitor”, although how exactly this suppression is achieved is completely unknown.

A molecular mechanism of how SEACIT/GATOR1 acts as a GAP has been addressed in several functional and structural studies, but a complete consensus of how exactly the GAP function is exerted has not yet been achieved. Indeed, in an initial study by the de Virgilio group, it was demonstrated that in *S. cerevisiae*, Iml1/Sea1 can co-precipitate with Gtr1 in the presence but substantially less in the absence of other SEACIT subunits. Yet, in the in vitro binding and GAP essays, Iml1/Sea1 could directly bind to Gtr1 and promote GTP hydrolysis in the absence of Npr2 and Npr3. GAPs often supply a catalytic amino acid residue (Arg, Asp or Gln) in their active sites, thus forming an “arginine finger” or “Gln/Asn thumb” that can be inserted into nucleotide-binding pocket of a GTPase [109]. In the highly conserved Iml1/Sea1 domain, essential for its GAP activity (aa 929-952), a conserved Arg^943^ was critical for GAP activity both in vitro and in yeast cells. Human DEPDC5 could partially complement TORC1 inhibition defect in *iml1Δ* cells, suggesting a conserved role of Iml1/Sea1 and DEPDC5 across the species. Therefore, when the cryo-EM structure of GATOR1 (Figure 2A) and GATOR1 in the complex with RAG GTPases was solved, it came as a surprise because it revealed a very unexpected mode of interaction between GTPases and GAPs [29].

For the structural studies, GATOR1 was copurified with RAG GTPase heterodimer, containing wild type RAGA and mutant RAGC, which can bind GTP, but not GDP. In addition, this heterodimer was loaded with GDP and non-hydrolysable GTP analogue (GppNHp) to create the most favorable nucleotide-binding configuration for interaction with GATOR1. The structure demonstrated that the overall conformation of the GATOR1 in a complex with RAG GTPases is similar to a free GATOR1 (see above). The SHEN domain of DEPDC5 can contact directly with a site proximal to nucleotide binding pocket of GTP analogue-bound RAGA. However, quite surprisingly, this interaction did not appear to be responsible for the stimulation of GTP hydrolysis. The kinetic analysis of GTP hydrolysis of DEPDC5 alone with RAGA or NPRL2/NPRL3 dimer with RAGA revealed that it is rather NPRL2/NPRL3, which has GAP activity. Moreover, a conserved Arg^78^ localized on the loop of NPRL2 longin domain is the “arginine finger”, responsible for GAP activity [110]. However, this Arg^78^ is located far away and is opposite to the RAGs binding interface of DEPDC5. Moreover, an earlier study from Wang laboratory showed that amino acid stimulation enhances the interaction of RAGA with both endogenous DEPDC5 and NPRL3 [111]. To explain these rather contradictory observations, a two-state model of GATOR1 interaction with RAG GTPases was proposed: in the inhibitory mode, DEPDC5 SHEN domain interacts strongly with RAGs and GAP activity of GATOR1 is weak; alternatively, a low affinity interaction dependent on NPRL2/NPRL3 stimulates GAP activity. Such bi-modal activity has not been previously observed between a GAP and a GTPase. Moreover, before this study, longin domains were found to be highly represented in many GEFs, where they would serve as adaptable platforms for GTPases [42]. In addition, in a structure of *Chaetomium thermophilum* Mon1-Ccz1-Ypt7 complex, Mon1-Ccz1 GEF contacts its cognate GTPase Ypt7 through a face of a conserved longin domain heterodimer [112]. NPRL2 and NPRL3 also form a heterodimer using their longin interaction domains; therefore, it is quite intriguing why in case of Mon1-Ccz1 longin heterodimer supports a GEF activity, while NPRL2/NPRL3 longin domains assist to GAP function. One of the plausible explanations might involve a possibility that NPRL2–NPRL3 interaction with RAGs can be sterically compromised by GATOR2, because it is NPRL3, which is necessary and sufficient for interaction with GATOR2. Finally, to add even more complexity, one (and the only) study reported that NPRL2 interacts with RAGD in amino acid scarcity, and with Raptor during amino acid sufficiency to activate mTORC1 [113]. Although the authors explain this behavior by suggesting that NPRL2 may not solely exist as a part of GATOR1, these findings require more clarifications.

It is evident that more structural studies will be necessary to explain this peculiar mode of interaction between GATOR1 with RAG GTPases. For example, a structure of RAGs-NPRL2-NPRL3 would allow to observe the conformation of the active GAP, a task that will not be very easy, given a weak association of NPRL2/NPRL3 heterodimer with RAGs in the absence of DEPDC5. In addition, solving a structure of yeast SEA complex, where the association between SEACAT (GATOR2) and SEACIT (GATOR1) is much stronger and where GAP activity seems to be performed by Iml1/Sea1 (DEPDC5), rather than by other components of the complex, would be absolutely central for the elucidating how SEACIT/GATOR1 exert its GAP function.

### 5.6. SEA/GATOR Recruitment to the Vacuolar/Lysosomal Membrane

In yeast, both TORC1 and SEA complex localize at the vacuole membrane regardless of the presence or absence of amino acids [3,77,114,115]. Iml1/Sea1 did not require other SEA components to localize to the vacuole membrane in both budding and fission yeast [11,28]. In contrast, Npr2 and Npr3 mutually depend on each other and on Iml1/Sea1 for vacuolar localization [11,28]. Importantly, the deletion of any of the SEACIT components during nitrogen starvation caused the re-localization of Tor1 to the cytoplasm [13].

In mammalian cells, mTORC1 is recruited to the lysosome in the presence of the amino acids, where it is fully activated by RHEB [116]. In addition, the activation of mTORC1 by RHEB can happen at the surface of other organelles, because both RHEB and mTORC1 have been detected at the Golgi apparatus, the peroxisome, the plasma membrane and ER [62,117,118]. Stably expressed GFP-tagged components of GATOR1 (NPRL2 and DEPDC5) and GATOR2 (MIOS and WDR24) localize to the lysosome regardless of the amino acid levels [12,67], although a recent study revealed that during amino acid starvation, WDR24, MIOS and mTOR can be found at a rough ER membrane [119]. Similarly, *Drosophila* GATOR2 components Mio and Seh1 localize to lysosomes in both fed and starved flies. Mammals, however, developed additional mechanisms to maintain GATORs at the lysosomal membrane, which include an interaction with the protein complex KICSTOR, that is not present in non-vertebrates and the regulation of GATOR1-RAGA interaction via ubiquitination.

The mammalian-specific KICSTOR complex identified in 2017 plays a key role in the localization of GATOR1 to its GTPase substrates [66,67]. KICSTOR consist of four proteins, KPTN, ITFG2, C12orf66 and STZ2, whose initial letters gave the complex its name. *C. elegans* only encode a homologue of SZT2, while yeasts and *Drosophila* lack entire KICKSTOR [15,67]. Both GATOR1 and GATOR2 associate with KICKSTOR in an amino-acid insensitive manner. STZ2 is responsible for the interaction of KICKSTOR with GATOR1, since STZ2 knockouts impaired the localization of GATOR1 to the lysosomes, but not GATOR2 or RAG GTPases. SZT2 is also necessary for the coordinated GATOR1 and GATOR2 binding and for GATOR1-dependent inactivation of mTORC1 at the lysosome. SZT2 contains several regions that allow interaction with GATOR1 and GATOR2 [66]. SZT2–DEPDC5 interactions can occur in the absence of other GATOR components [29]. SZT2 does not bind GATOR2 in the absence of NPRL3, once again underlining a crucial role of this protein in GATOR1–GATOR2 interactions. In addition, lysosomal localization of WDR59 is abolished in the absence of SZT2. Thus, KICKSTOR, and especially its largest component 380 kDa SZT2, may facilitate interaction between GATOR1 and GATOR2 and maintain both subcomplexes together. In contrast, in *S. cerevisiae*, both SEA subcomplexes can form a stable complex without other mediating proteins. It is intriguing why, during evolution, mammals acquired a large protein complex to maintain interactions between GATOR1 and GATOR2, which otherwise are quite stable in lower eukaryotes.

GATOR1 is also implicated to the recruitment to the lysosomal surface of another GAP—FLCN/FNIP. GATOR1-dependent control of the RAGA nucleotide state drives FLCN recruitment to lysosomes when amino acids are scarce [120]. Indeed, when amino acids are low, the GAP activity of GATOR1 promotes the GDP-RAGA/B conformation and FLCN/FNIP is recruited to the lysosome to act as a GAP towards RAGC/D. In this study, only knockout of *NPRL3* in HeLa cells were verified, and it is not known whether knockout of other GATOR1 components would have the same effect. Nevertheless, these findings help to resolve the apparent contradiction reported earlier, that FLCN-FNIP heterodimer binds to RAGA/B, but acts as a GAP for RAGC/D [69,121]. Cryo-EM structures of the human FLCN-FNIP-RAG-RAGULATOR complex containing an inactive form of the RAG heterodimer confirmed that the FLCN-FNIP2 heterodimer binds to the GTPase domains of both RAGA and RAGC [122,123].

GATOR1-RAGA interactions are controlled by several kinases and E3 ubiquitin ligases, which are not present in lower eukaryotes. For example, an oncogenic non-receptor tyrosine kinase, SRC, disrupts GATOR1-Rags interactions, promoting mTORC1 recruitment and activation at the lysosomal surface [124]. Currently, it is not known what the mechanisms that activate SRC in response to amino acids are and whether GATOR1 subunits or RAGs can be phosphorylated by SRC. On the other hand, DEPDC5 can be phosphorylated by Pim1 kinase at S1002 and S1530, and by AKT also at S1530 [125]. This phosphorylation seems not to affect the ability of DEPDC5 to interact with neither NPRL2 nor SZT2, but elevated Pim1 expression during amino acid starvation overcame mTORC1 suppression.

Two lysosome localized E3 ligases, RNF152 and SKP2, mediate K63-linked polyubiquitination of RAGA at different sites, which promote GATOR1 recruitment to RAGA and the consequent inactivation of mTORC1 [111,126]. Remarkably, SKP2 ubiquitinates RAGA at K15 during prolonged amino acid stimulation [126], while, quite opposite, RNF152 ubiquitinates RAGA at a different set of lysines (K142, 220, 230, 244) during amino acid starvation [111]. SKP2 provides a negative feedback loop, where RAGA ubiquitination and GATOR1 recruitment restrict mTORC1 activation upon sustained amino acid stimulation. Inversely, during amino acid starvation, it is RNF152-dependent RAGA ubiquitination, which enhances GATOR1–RAGA interaction. Interestingly, RNF152 can also ubiquitinate RHEB, sequestering RHEB in its inactive RHEB-GDP form and promoting its interaction with TSC2, which leads to mTORC1 inactivation [127]. Thus, RNF152 acts a negative mTORC1 regulator in both amino acid and growth factor brunches of mTORC1 signaling.

### 5.7. SEA/GATOR in Autophagy

One of the major functions of mTORC1 is in the regulation of autophagy, which is induced when mTORC1 is inhibited. Thus, it is not surprising that deletions of SEACIT/GATOR1 components suppress autophagy in yeast [3,9,13,103,104,128,129], *Drosophila* [130], *C. elegans* [131] and mammals [129,132]. Just as the opposite, mutations in GATOR2 may promote autophagy, which can happen even in the absence of nutrient starvation, as it is a case of *wdr24* mutants in *Drosophila* [133]. In contrast, deletions of SEACAT members in yeast seem not to have a drastic effect on autophagy initiation and flux [3]. Interestingly, the nitrogen starvation deletion of *SEA1* or double deletion of *NPR*2 and *NPR3* resulted in the inhibition of vacuolar fusion [13]. As the inactivation of TORC1 during nitrogen deprivation promotes vacuole coalescence [134], deletions of any of the SEACIT members increase TORC1 activity during starvation, and, therefore, induce vacuolar fragmentation and defects in autophagy.

Recently a bi-directional feedback loop, which regulates autophagy and involves SEACAT, has been described [50]. In order to control autophagy, TORC1 phosphorylates and inhibits the Atg1 kinase essential for autophagy initiation, but Atg1, in turn, can phosphorylate SEACAT components. Although it is currently not known whether that phosphorylation acts positively or negatively on TORC1 activity, this finding uncovers the important node of convergence between TORC1 and Atg1, with the SEACAT being both the regulator and effector of autophagy.

**Figure 4:**
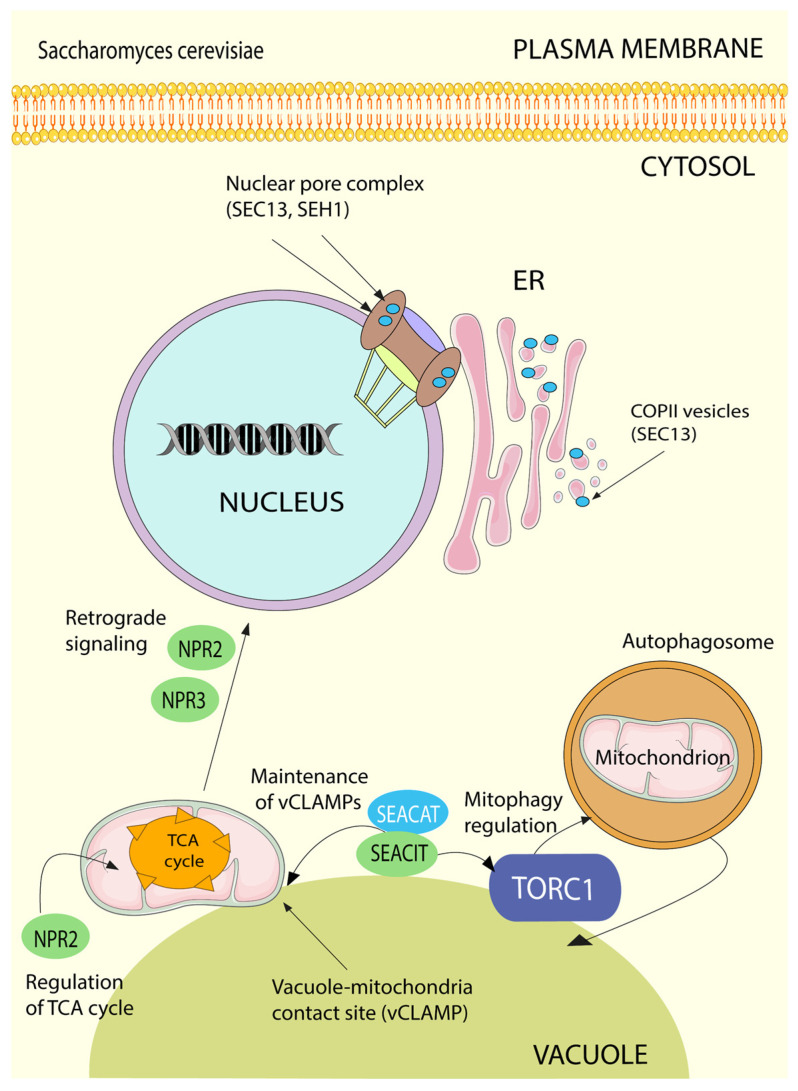
Functions of the *S. cerevisiae* SEA complex and its components beyond nutrient response. Indicated are Seh1 and Sec13 as components of the nuclear pore complex and Sec13 as part of COPII vesicles. Npr2 and Npr3 regulate retrograde signaling. Npr2 is also involved in the regulation of TCA cycle. Finally, SEA complex is involved in the maintenance of the vacuole-mitochondria contact sites (vCLAMPs) and is important for mitophagy.

The SEA complex is also important for specific types of autophagy. Thus, yeast with deletions of SEACIT complex members failed to activate selective degradation of mitochondria via mitophagy (Figure 4) [135,136]. Given the conservation of the SEA/GATOR function, it is reasonable to assume a similar role of GATOR in mammals, although the involvement of GATOR in specific types of autophagy in mammals has not yet been described.

## 6. SEA and GATOR Functions beyond Nutrient Responding

### 6.1. SEA/GATOR Evolution Origin

**Figure 5:**
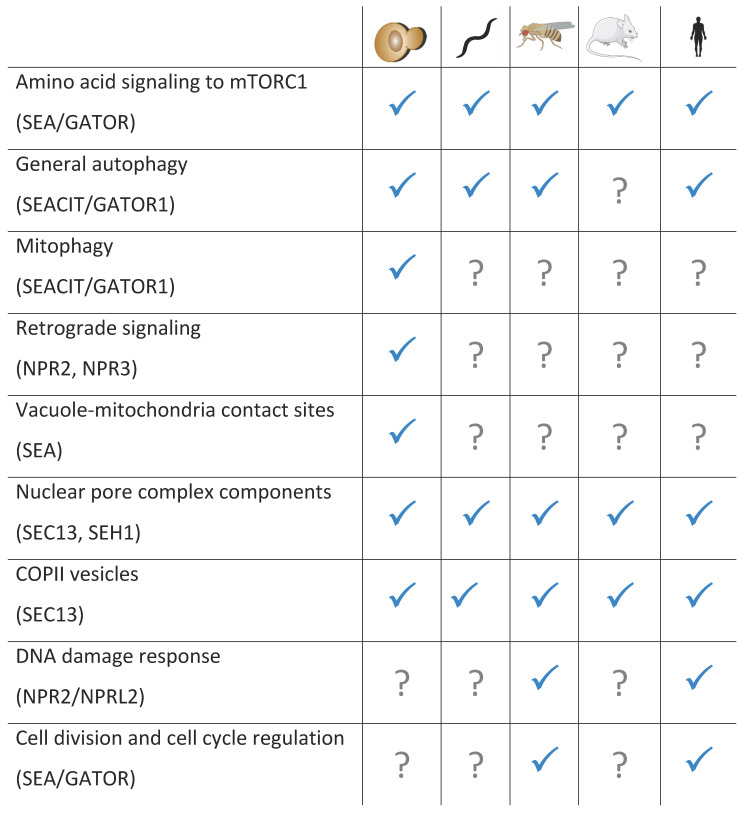
Multiple functions of the SEA/GATOR complex.

SEA/GATOR has always been “living double lives” with a number of its components having diverse “moonlighting” functions beyond their role in the regulation of nutrient sensing and responding (Figure 4 and Figure 5). Although the majority of these functions seem to be related to the SEA/GATOR role in the regulation of mTORC1, others are clearly associated with totally different pathways. Accordingly, despite the fact that the main localization site of SEA/GATOR is a vacuole/lysosomal membrane, some of its components can be found in the nucleus, ER, mitochondria, plasma membrane, etc., depending on the functions that they fulfil in different cell types, stages of cell cycle progression and physiological conditions [24,54,94,135,137,138,139,140]. The most outstanding examples are Seh1 and Sec13, which together are the members of the Nup84 subcomplex in the nuclear pore complex, with Sec13 also being a component of COPII coated vesicles [3]. This “double life” of Seh1 and “triple life” of Sec13 witnesses the evolution of the endomembrane system. Indeed, the progression from prokaryotic to eukaryotic cells was accompanied by the acquisition of membranous structures, eventually transformed into organelles, which often adopted preexisting molecules and adjusted them for new needs via duplication and neofunctionalization [33]. During this transformation, a central role was played by ancient protocoatomers, which facilitated membrane bending. Not only Seh1 and Sec13, but the entire SEACAT/GATOR2 complex belongs to the large family of protocoatomer-derived complexes that form transport vesicles (COPI, COPII, clathrin), membrane-associated coats (nuclear pore complexes), tethering complexes (HOPS/CORVET) and other membrane associated structures, such as SEACAT/GATOR itself [3,31,34]. These various assemblies have a number of structural similarities, including a hallmark feature—a presence of N-terminal β-propeller, formed by WD40 repeats, and C-terminal α-soleniod composed of α-helices (HEAT repeats). In that view, Sea4/MIOS is the most well preserved protocoatomer descendant, while Sea2/WDR59 and Sea3/WDR24 diverged more profoundly, loosing many α-helices, but still preserving N-terminal β-propellers.

GTPases, with their corresponding GEFs and GAPs, are other important elements of membrane-associated assemblies. SEACIT/GATOR1 carries this functional feature of endomembrane system, being a GAP for RAGA GTPase. In addition, longin domains present in two components of the SEACIT/GATOR1 can also be found in small GTPases and many other proteins involved in assembly, fusion and tethering of membranes [141]. Here, again, paralogs Npr2/NPRL2 and Npr3/NPRL3 evidence that evolution progressed through duplication and divergence, because both proteins seem to have additional functions, apart from mTORC1 regulation.

Remarkably, the entire vacuole/lysosome-associated mTORC1 pathway machinery contains multiple structural elements typical for classical endomembrane systems [30]. For example, the mTORC1 complex has a β-propeller subunit mLST8, structurally very close to Seh1 and Sec13. Similar to other coatomers, another mTORC1 subunit, Kog1/RAPTOR, contains HEAT repeats and β-propeller, but in a “Lego game of evolution” these structural elements switch places with HEAT repeats situated at the N-terminus and β-propeller at the C-terminus. By the way, in the mTORC1 complex, RAPTOR interacts with the HEAT domain of mTOR. Finally, the abundance of small GTPases, GAPs and GEFs that control mTORC1 witness the common evolution origin of the core endomembrane system and its regulators.

### 6.2. Regulation of Mitochondrial Biogenesis and Quality Control

The mTORC1 pathway plays an essential role in mitochondrial biogenesis, mitochondrial genome repair, the phosphorylation of mitochondrial proteins and the regulation of mitophagy, the selective degradation of mitochondria by autophagy. As a central controller of the mTORC1 pathway, SEA/GATOR is also involved in the regulation of mitochondria function and quality control (Figure 4, Figure 5). The analysis of synthetic genetic interactions in *S. cerevisiae* revealed already in 2011 that SEA genes interact with many mitochondrial genes, with Npr2 located close to the mitochondrial gene cluster [3,142,143]. About 20% of proteins that co-precipitate with SEA components are mitochondrial proteins [13,135] and, inversely, enriched mitochondrial fractions contain SEA proteins [137]. Both C-terminal GFP tagged Iml1/Sea1 and Sea4 can be localized to the mitochondria [135]. Moreover, treatment with rapamycin significantly increases the amount of cells with cytoplasmic and mitochondrial localizations of Iml1/Sea1, although a fraction of Iml1/Sea1 can still be observed at the vacuole [138]. Similarly, in HEK 293T cells NPRL2 can be localized to the mitochondria and many mitochondrial proteins can be found in the proteome of NPRL2 and NPRL3 [54]. Recently, SESTRIN2, which interacts with GATOR2 during leucine starvation (see above), was also found to be localized to mitochondria and silencing of GATOR2 genes considerably reduced the mitochondrial pool of SESTRIN2 [144]. Finally, Sec13 was shown to be interacting with mitochondrial antiviral signal protein (MAVS, also known as VISA) [145,146]. MAVS is localized on the outer membrane of mitochondria, with a small proportion present at mitochondria-associated membranes (MAMs). Sec13 overexpression increases MAVS aggregation and facilitates interferon β production, while low levels of Sec13 result in a weaker host antiviral immune response. Currently, it is not clear whether other proteins from nuclear pore complex or COPII or GATOR2 are also involved in these interactions.

The deletion of SEA/GATOR components affects mitochondria functions. The total abundance of SEA proteins is increased during respiratory growth and decreased upon nitrogen starvation, *sea2* deletion impairs respiration capacity in *S. cerevisiae* [147]. *npr2Δ* cells have defective mitochondrial-housed metabolic pathways, such as synthesis of amino acids, and an impaired tricarboxylic acid (TCA) cycle activity. *npr2-*deficient cells showed decreased pools of nitrogen-containing intermediates of the TCA cycle and nucleotides. Yet, *npr2Δ* yeast use TCA cycle intermediates for replenishment of biosynthetic pathways to sustain the hypermetabolic state due to mTORC1 constant activation, suggesting a role of SEACIT in the regulation of cataplerotic reactions of the TCA cycle depending on the amino acid and nitrogen status of the cell [148]. This was later supported by another study that demonstrated that skeletal-muscle-specific NPRL2 loss in mice promoted aerobic glycolysis by altering the tuning between the amino acid sensing pathway and TCA cycle function. NPRL2-mKO mice also had less oxidative muscle fibers and more glycolytic muscle fibers, a hallmark of aerobic glycolysis, which highlights the functional role of NPRL2 in vivo in the regulation of glucose entry into the TCA cycle [149].

The function of GATOR1 proteins in mitochondrial health seems not to be limited to NPRL2. A heterozygous mutation in the CTD domain of DEPDC5 gene found in an autistic child was correlated with a significant decrease in mitochondrial complex IV activity and decrease in the overall oxygen consumption rate in peripheral blood mononuclear cells. Therefore, this variant of DEPDC5 can be directly related to an altered mitochondrial function in autistic disease [150]. Mice with skeletal-muscle specific deletion of DEPDC5 showed increased mitochondrial respiratory capacity and TCA cycle activity [151].

SEACIT is also involved in the communication of the mitochondria with other organelles. The mitochondria-to-nucleus communication pathway, known as the retrograde signaling, is triggered by mitochondrial dysfunctions in order to alter the expression of nucleus-encoded mitochondrial genes to effect metabolic reprogramming and to restore cellular fitness [152,153]. *npr2∆* and *npr3∆* yeast strains failed to activate the retrograde signaling pathway when grown in media containing ammonia as nitrogen source [10,148]. In order to recruit the substrates for biochemical reactions and export resulting products mitochondria rely on direct transport with organelles through contact sites [154]. The vacuole and mitochondria contact sites, vCLAMPs, are important for lipid exchange [155] and may also serve for the sensing of the integrity and functionality of mitochondria (Figure 4) [135]. Importantly, SEACIT is required for the maintenance of vCLAMPs and the deletion of any SEACIT members drastically reduces the amount of vCLAMPs in yeast cells [135]. Whether GATOR1 has the same functions in mammalian cells remains to be discovered.

### 6.3. GATOR1 and DNA Damage Response

The notion that Npr2/NPRL2 might have a role in DNA damage response appeared when it was found that mutations in this protein, both in yeast and human, confer resistance to the anticancer drugs cisplatin and doxorubicin (Figure 4 and Figure 5) (see below) [156,157]. These compounds induce high levels of DNA damage, which eventually lead to cell cycle arrest and apoptosis [158,159]. Study of the role of NPRL2 in DNA damage response in non-small-cell-lung cancer cells treated with cisplatin [160] demonstrated that the ectopic expression of NPRL2 activates the DNA damage checkpoint pathway in cisplatin-resistant and NPRL2-negative cells, leading to cell cycle arrest in the G2/M phase and induction of apoptosis. Upon ectopic expression, NPRL2 promotes ROS production via NADPH oxidase (NOX) 2 activation [54]. Overexpressed NPRL2 accumulates in the nucleus, where it interacts with the apoptosis initiation factor, AIF. In addition, NPRL2 expression provokes the phosphorylation of tumor suppressor p53, which, in turn, activates a DNA-damage checkpoint pathway via p21 and CDC2. An excessive amount of NPRL2 results in cell cycle arrest in G1 phase in cells with constitutively p53 and to CHK2-dependent S or G2/M in p53-negative cancer cell lines [54,161]. Currently, it is not known whether these functions are performed by NPRL2 as a part of GATOR1 complex, or separately. *Drosophila* GATOR is also critical to the response to meiotic double strand DNA breaks (DSB) during oogenesis, since depletion of each GATOR1 component fails to repair DSB with nprl3 mutants showing increased sensitivity to genotoxic stress both in germline and somatic cells [162].

### 6.4. GATOR in Cell Division and Cell Cycle Regulation

GATOR2 is important for both mitotic and meiotic division (Figure 5). Depletion of MIOS in HeLa cells resulted in mitotic defects, such as spindle assembly defects and delay or failure in cytokinesis [163]. MIOS regulates mitotic events through Aurora A kinase and Polo-like kinase 1 (Plk1), which control the localization and function of mitotic spindle. MIOS is important for spindle formation, subsequent chromosome segregation and proper concentration of active Plk1 and Aurora A at centrosomes and spindle poles. SEH1, which forms a complex with MIOS (see above), targets GATOR2 to mitotic chromosomes, required for the localization of chromosomal passenger complex and functions in chromosome alignment and segregation by regulating the centromeric localization of Aurora B [164]. This function of GATOR2 nevertheless seems to be related to its role in mTORC1 activation, because depletion of MIOS causes reduced mTORC1 activity at centromeres in mitotic cells [163].

In *Drosophila*, Mio localizes to oocyte nucleus at the onset of prophase and meiosis I, and is required for the maintenance of the meiotic cycle during oocyte maturation [24]. *Drosophila* Seh1 is also involved in the maintenance of meiotic cycle and regulation of microtubule dynamics in ovarian cysts [36]. Depletion of *iml1* in the female germ line delays mitotic/meiotic transition and ovarian cysts undergo an extra mitotic division [18]. Thus, GATOR1 downregulates TORC1 activity to promote the mitotic/meiotic transition in ovarian cysts, while inhibition of GATOR1 by GATOR2 prevents the constitutive downregulation of TORC1 at the later stages of oogenesis.

### 6.5. The Role of GATOR in Development

Animal development and growth is closely related to the ability to respond to different nutrient cues. Therefore, it is not surprising that GATOR components are important at different stages of embryonic and somatic development. Various studies in *Drosophila* by Lilly’s group demonstrated that mutations of *mio*, resulting in the production of truncated protein, suppresses oocyte growth and differentiation [24]. Seh1 in *Drosophila* is also required in oogenesis, but is dispensable for somatic development [36]. Both Mio and Seh1 promote TORC1 activation in female fly’s germ lines, but play a relatively minor role in the activation of TORC1 in many somatic types [18]. Wdr24, which is also required for ovary growth and female fertility, promotes TORC1-dependent cell growth not only in germ line, but also in somatic tissues of *Drosophila* [133]. *nprl2* mutations in *Drosophila* decrease the lifespan in flies, which have an accelerated gastrointestinal tract aging process [165].

In *C. elegans,* NPRL2 and NPRL3 are required for postembryonic development, which is supported by the availability of a specific sphingolipid. When *C. elegans* larvae are placed in the environment lacking this lipid, they suspend growth and cell division, which can be overcome by resupplying the lipid. When this lipid is absent, postembryonic growth and development can be re-initiated by activating TORC1 or inhibiting NPRL2/3 [17]. NPRL3 represses intestinal TORC1 activity at least in part by regulating apical membrane polarity, which is probably the main reason of larval development defects in worms that are not supplied with a sphingolipid [166] In addition, *nprl3*-deficient worms grow slowly due to the lack of the ability to sense vitamin B2 deficiency in their food [131]. NPRL3 deficiency in worms’ intestines triggers a gut protease activity, which derives in abnormal behavior and growth impairing [131].

## 7. Deletion Phenotypes of the SEA/GATOR Components across Different Species

In unicellular yeast *S. cerevisiae*, SEA genes (apart from Sec13) are non-essential [3] and in rich media, SEA deletion mutants grow practically with the same rate as wild type yeast [3]. In fission yeast *S. pombe*, deletion of any GATOR1 as well as GATOR2 component Sea3 results in a severe growth defect [22,28]. Homozygous deletions of *nprl2* and *nprl3* in *Drosophila* are semi-lethal and deletions of *iml* are lethal, with GATOR1 activity required for animals to transit the last stage of pupal development [130]. In addition, *nprl2* null flies have a significantly reduced lifespan [165]. Similarly, *depdc5* knockout in zebrafish resulted in premature death at 2–3 weeks post-fertilization [167]. In mice homozygous knockouts of Seh1 [168], Wdr59 and Wdr24 are embryonically lethal [169]. Constitutive knockout homozygous and heterozygous GATOR1 rodent models differs significantly. Thus, GATOR1 homozygous animals *Nprl2^−/−^* mice [20], *Nprl3^−/−^* mice [41], *Depdc5^−/−^* rats [21] and *Depdc5^−/−^* mice [170] are embryonically lethal. Mice embryos deficient for NPRL2 expression show a compromised liver hematopoiesis, which has a negative impact on embryonic viability [20]. Although mutations in GATOR1 genes are associated with epileptic disorders and brain malformations, heterozygous *Depdc5^+/−^* rats and mice did not present spontaneous epileptic seizures, but *Depdc5^+/−^* rats have subtle cortical malformations [21,170]. Several tissue specific knockouts have also been investigated. Neuron-specific conditional homozygous Depdc5 knockout mice lived till adulthood, but had larger brains and exhibited a decreased survival [171]. The hepatic deletion of *Depdc5* in mice resulted in mild liver inflammation and decreased fat level [172]. Skeletal muscle-specific Depdc5 depletion in mice resulted in muscle hypertrophy, but neither the physical nor contractile muscle function of these mice improved [151]. Similarly, mice with Nprl2 deletion in skeletal muscles had larger muscle fibers and exhibited altered running behavior [149]. In conclusion, deletions of SEA/GATOR components in every organism studied thus far provoked severe defects on growth and viability.

## 8. GATOR in Human Diseases

**Figure 6:**
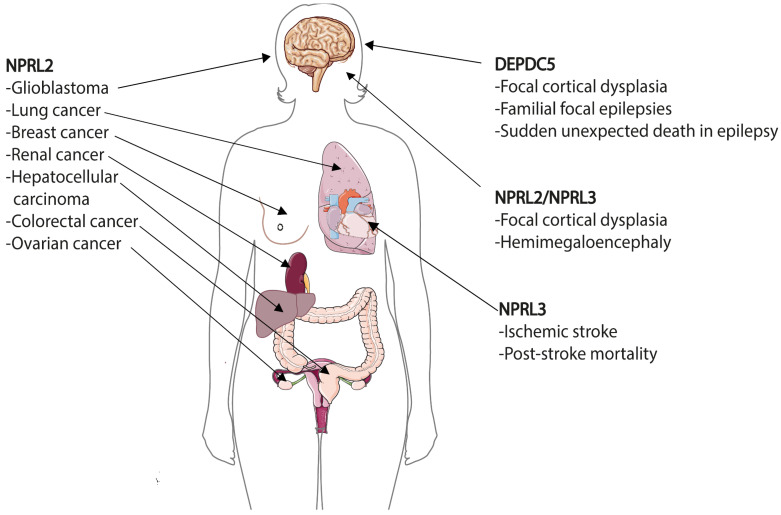
Deregulation of GATOR1 components in different human diseases. Expression of GATOR1 components is downregulated in many cancers and GATOR1-related neurological disorders.

During the last decade it became increasingly evident that alternations in the expression of GATOR genes can cause various human diseases (Figure 6). Mutations of GATOR2 components can be found in various cancers according to The Cancer Genome Atlas (TCGA) and Cancer Cell Line Encyclopedia (CCLE), COSMIC and cBioPortal databases, although their recurrent mutation frequency is very low [173]. None of the GATOR2 mutations in these cancers were studied on the molecular level and currently there are no data about the involvement of GATOR2 components in other human pathologies [174]. One of the reasons of the low pathogenicity of GATOR2 mutations could be that they would cause an increased, but most probably not complete, suppression of the mTORC1 pathway, which can rather be associated with healthier conditions.

In striking contrast to GATOR2, many pathological mutations in GATOR1 genes have been reported. These mutations are mainly related with two main types of human diseases—cancer and epilepsy. Although the alternations in sequence and gene expression associated with these pathologies have been reported for all three GATOR1 genes, there are striking differences that mark some kind of “preferences” of a gene for a pathology. Thus, DEPDC5 mutations are more frequent in epilepsies in comparison with mutations in other GATOR1 members. NPRL2 mutations can be found more often in different types of cancers and are associated with resistance to anticancer drugs cisplatin and doxorubicin. Even though NPRL3 is a paralogue of NPRL2, its alternations in cancer are less recurrent. Instead NPRL3 appeared to be required for the normal development of the cardiovascular system. Below we will describe alternations of GATOR1 expression in different diseases.

### 8.1. Epilepsies and Brain Malformations—DEPDC5 and Others

In 2013, DEPDC5 was reported as the first gene implicated in familial focal epilepsies by Baulac and Scheffer groups [175,176]. In the following years, it became clear that mutations in DEPDC5 are also related with brain malformations, notably with focal cortical dysplasia (FCD), which is a major cause of drug-resistant epilepsy [177] and can be associated with sudden unexpected death in epilepsy (SUDEP) [178]. In 2016, mutations related with focal epilepsies, familial cortical dysplasia and SUDEP were also reported for Nprl2 and Nprl3 [174,179,180]. Since then, more than 140 variants of GATOR1 genes have been found in up to 37% of patients with familial focal and in other forms of epilepsies [181]. These variants include loss-of-function mutations (67%), missense mutations (27%), splice site changes (4%), frameshifts and copy number variants (~1%). Interestingly, the distribution of mutations in an epilepsy cohort differs drastically from the overall distribution of GATOR1 mutations listed in the gnomAD database, where loss-of-function represents only 4% of variants, with the majority (88%) being missense mutations. Importantly, histopathological analysis of brain tissues resected from individuals with GATOR1 gene mutations demonstrate the hyperactivation of mTORC1 pathway, suggesting that mTORC1 signaling plays an important role in brain development [174,179,180].

Nearly 85% of GATOR1 mutations in epilepsies account for changes in DEPDC5 with both somatic and germline mutations detected all through the gene without clustering. Initially, it was not clear how germline *Depdc5* mutations can cause FCD, especially taking into account that these mutations are often dominantly inherited from an asymptomatic carrier parent [181] and that in rodent models *Depdc5^+/−^* constitutive heterozygous mutations do not exhibit an epileptic phenotype [21,170]. The discovery of second hit somatic mutations in trans, which led to a biallelic inactivation in a subset of brain cells, explained this phenomenon [182,183]. Nprl2 and Nprl3 mutation are less frequent (6% and 9%, respectively), which might be partially related with the fact that their involvement in epilepsies and brain malformations has been tested in a low number of people [26,181]. Cases with simultaneous mutations in different GATOR1 genes have not been described thus far. Several *Nprl2* or *Nprl3* variants found in individuals with FCD or hemimegaloencephaly (HME) have been reported recently [184,185]. Interestingly, *NPRL3* single nucleotide polymorphism has been associated with ischemic stroke susceptibility and post-stroke mortality [186], which can be related with increased mTOR activity, that is known to accelerate brain recovery after stroke. The role of NPRL3 in this disease is most probably related with its function in focal epilepsies that might occur in ischemic cerebrovascular disorders [187]. Finally, genetic alternations of KICSTOR complex, required for GATOR1-mediated repression of mTORC1 signaling (see above), have also been linked to epilepsies and brain malformations [188,189,190].

Thus, it is evident that GATOR1 plays an essential role in cortical formation and development. Mutations of GATOR1 components became important features of “mTORopathies”—a set of pathological conditions characterized by brain malformations, neurological disorders and mTORC1 hyperactivity due to either gain-of-function mutations in a pathway activators (e.g., *AKT*, *RHEB*, *MTOR* itself) or loss-of-function mutations of inhibitors (e.g., *TSC1, TSC2*) [191,192]. However, mutations of GATOR1 genes seem to result in a broader spectrum of neurological disorders than other “mTORopathic” genes. Not only are these mutations highly related with medically intractable epilepsies and, especially SUDEP, but they are also observed in autism spectrum disorders [150] and could be implicated in Parkinson’s disease [193]. Therefore, it was recently proposed to name GATOR1-related neurological disorders as GATORopathies [194].

### 8.2. Cancer and Anticancer Drug Resistance—NPRL2 and Others

Among GATOR1 components, NPRL2 was the first that was suggested to be a tumor suppressor [195] almost a decade before the GATOR1 complex was described for the first time. NPRL2 has the higher cancer-associated recurrent mutational frequency out of all the GATOR1 genes [173]. For example, missense mutations in metastatic breast cancers are twice more frequent in Nprl2 (1.55%), than in Nprl3 or Depdc5 (0.78%) [196]. Low levels of NPRL2 expression have mostly been detected in solid tumors (Figure 6), including hepatocellular carcinoma [197], glioblastoma [12], as well as in renal [198,199], ovarian [12,199], colorectal [199,200,201,202], breast [199,203] and lung cancers [157,160,199,204,205]. Paradoxically, NPRL2 might also have functions as an oncogene. Recent studies in castration resistant prostate cancer (CRPC) revealed that poor prognosis is associated with high expression of NPRL2 [206].

Alternations of NPRL2 expression is also related to the resistance to a number of anticancer drugs. The most recurrent cases are associated with the resistance to cisplatin and doxorubicin, which has been initially observed in Npr2 deletion mutants in yeast [156] and further confirmed in human lung cancer cell lines [157,160]. The reason of this resistance is still not clear, but it could be related with a role of NPRL2 in DNA damage response (see above) [54,160]. Overexpression of NPRL2 in colon cancer cells increases the sensitivity to a topoisomerase I inhibitor irinotecan (CPT-11) by activation of the DNA damage checkpoints [207]. Genomic alternations of all three GATOR1 components have recently been associated with the resistance to PI3Kα inhibitors in primary and metastatic breast cancer [208]. This resistance is explained by the sustained activation of the mTORC1 pathway due to the loss of function mutations of GATOR1 components. In this case, it is reasonable to expect that concomitant mTOR blockage by rapalogs or mTOR pan-inhibitors might overcome resistance. Inversely, CRPC cells, where NPRL2 expression is elevated, are resistant to everolimus [209].

Surprisingly, during the last decade, not a single article reported a study about the involvement of NPRL3 in cancer and drug resistance, even if in the COSMIC database there are almost three times more somatic cancer mutations listed for NPRL3 than for its paralogue NPRL2.

A low frequency DEPDC5 inactivation mutation has been observed in glioblastoma and ovarian cancer, but was not further investigated [12]. DEPDC5 downregulation was also observed in tumors of breast cancer patients [53], where it is strongly correlated with the upregulation of KLHL22 E3-ubiquitin ligase, responsible for DEPDC5 polyubiquitylation and degradation (see above). Recently, DEPDC5 inactivation was discovered in gastrointestinal stromal tumors (GIST), one of the most common human sarcomas. Chromosome 22q deletions are observed in ~50% of GIST and recurrent genomic inactivation of DEPDC5 (>16%) makes it the bona-fide tumor suppressor contributing to GIST progression via increased mTORC1 pathway signaling [210]. This is in striking contrast with >250 non-GIST sarcomas where DEPDC5 aberrations are infrequent (~1%). Interestingly, cancer occurrence in epilepsy probands with germline GATOR1 variants is very low and at present it is considered that there is no link between epileptic germline GATOR1 variants and cancer [181].

Currently, >2000 somatic mutations in different tumors are listed for GATOR1 genes in the COSMIC database, none of them have been studied in detail. It is reasonable to expect that in the following years we should gain more information about the molecular mechanisms associated with the tumorogenesis provoked by these mutations.

### 8.3. Cardiovascular Diseases—NPRL3

In striking contrast to other GATOR1 components, and especially to its paralogue NPRL2, NPRL3 seems to be less important for epilepsy and cancer. Rather it appears as a crucial gene, necessary for the normal development of the cardiovascular system [41]. Mice with the deletion of NPRL3 promoter often have severe embryonal cardiac defects and die in late gestations. A single nucleotide polymorphism of NPRL3 was reported in sickle cell anemia [211], a disease characterized by various hemoglobin abnormalities. These defects are explained by the fact that the introns of NPRL3 contain super-enhancers required for high level expression of the genes encoding the α-globin subunits of hemoglobin in humans and mice [212,213]. These regulation elements appeared to be deeply preserved during evolution. A recent genomic study revealed that the NPRL3 gene carrying a strong regulatory element became linked to at least two different globin genes in ancestral vertebrate, just before the divergence between jawless and jawed vertebrates [214]. Each of these ancestral globin genes evolved in the modern hemoglobin genes, but kept their enhancers in NPRL3, which provide an explanation to a long-standing enigma of how globin genes linked to the same adjacent gene undergo convergent evolution in different species.

Therefore, the pathologies associated with NPRL3 mutations are related with the disturbances of the transcriptional elements in the Nprl3 gene rather than with the function of the protein product in the mTORC1 pathway. Similarly, the higher recurrence of NPRL2 mutations in cancers and DEPDC5 mutations in epilepsies could be related with the specific moonlighting functions of these GATOR1 members beyond the regulation of the mTORC1 pathway.

## 9. Conclusions

Since its discovery ten years ago, the SEA/GATOR complex has been recognized as an important regulator of the mTORC1 pathway that deals with the cell’s response to amino acid and glucose availability, DNA damage, mitochondria impairment, etc. Many studies have also revealed the role of the SEA/GATOR complex in human diseases, especially in cancer and epilepsies. Despite the growing number of discoveries involving the SEA/GATOR complex in many organisms, a lot of questions concerning its function and the mechanisms leading to pathologies are still left unanswered. For example, the role of the GATOR complex in amino acid sensing and response has been already clarified in great detail in several studies; however, it is still unknown whether the SEA complex in yeast can perform sensing functions, given that many amino acid sensors interacting with GATOR are not conserved in yeast. The functions of the SEA complex in autophagy and in the formation of organelle contact sites have been extensively studied in yeast. Whether the GATOR complex has these functions in higher eukaryotes is currently unknown (Figure 5). Finally, the most intriguing problem at the moment concerns the molecular function of the SEACAT/GATOR2 complex, an enigma that has remained unresolved despite these 10 years of research and discoveries. Without any doubt, having a high-resolution structure of this subcomplex with or without its partners (SESTRINs, CASTOR2 and others) will be crucial for understanding its function. It will be also important to figure out the principles of interaction between the two SEA/GATOR subcomplexes in different organisms, which can shed light on how evolution shaped this assembly to adapt for the particular needs of various species. SEA members appeared earlier than GATOR members, similar to crocodiles, which are slightly older than alligators [215]. In the same way with crocodiles and alligators, SEA and GATOR are similar to each other in terms of size, structure (appearance) and function (behavior). On the other hand, both SEA and GATOR have a number of subtle yet significant differences that might be able to explain how they each adjusted to operate optimally in different organisms and environments. For example, as with crocodiles, which are bigger than alligators, SEA components are also bigger than their human homologues. Therefore, it will not be surprising if the structural studies reveal that the shape of the SEA complex will slightly differ from that of the GATOR complex, as the V-shape crocodiles’ snout differs from larger U-shape snout of alligators. Despite the slight difference in shape, both reptiles use their snouts to effectively catch and hold the food. Similarly, SEA and GATOR complexes, despite several structural differences, can still respond to the presence of nutrients during regulation of the mTORC1 pathway. We are, therefore, confident that the next decade of SEA/GATOR research will lead to new exciting discoveries of the structure and function of this complex, that can better characterize its implication in health and diseases.

## Data Availability

Not applicable.
